# Design of Functional Fluorine-Containing Coatings for 3D-Printed Items

**DOI:** 10.3390/polym17212824

**Published:** 2025-10-23

**Authors:** Fedor Doronin, Georgy Rytikov, Andrey Evdokimov, Mikhail Savel’ev, Yuriy Rudyak, Victor Nazarov

**Affiliations:** Faculty of Printing Industry, Moscow Polytechnic University, 107023 Moscow, Russia

**Keywords:** surface design, additive manufacturing, FFF, ABS, PLA, TPU, PETG, filament, fluorination, wetting, friction, SEM, morphology

## Abstract

In this study, a surface texture design technique for 3D-extruded prototype products was developed. The study determines some target functional properties of polymer-made items. Four series of experimental samples (acrylonitrile–butadiene–styrene (ABS), thermoplastic polyurethane (TPU), polylactide (PLA), and polyethylene terephthalate glycol (PETG)) were 3D-printed using the fused filament fabrication (FFF) approach. The morphology and hydrophilic/hydrophobic balance of the surfaces of the experimental samples were regulated directly by the 3D design and by gas-phase fluorination techniques. The observed distilled water and ethylene glycol edge wetting angles of the surfaces of the experimental samples were determined by a 3D filament stroke arrangement. It was shown that varying the 3D design promoted hydrophobization and provided anisotropic wetting (the distilled water edge angle of the same sample varies from 76 to 116 degrees). The textured surfaces simultaneously demonstrated hydrophilicity in one direction and hydrophobicity in the other. The changing of the fluorine-containing gas mixture surface treatment duration allowed us to alter the hydrophilic/hydrophobic balance of 3D-extruded prototypes. The fluorination kinetics of the experimental samples were studied empirically. The combination of macroscopic surface design (through FFF 3D printing) and microscopic surface modification (through gas-phase fluorination) permitted a significant reduction in the straining friction coefficient and increased the wettability of the complex-shaped 3D-printed products.

## 1. Introduction

The creation of polymer-based materials with various special properties (heavy, conductive, and chemo- and/or bio-resistant) is one of the mainstream directions of research and development in the field of modern materials science and applied chemistry [1,2,3]. The corresponding products are designed to reduce the rate of biogrowth on marine vessels, for medical and pharmacological purposes, to seal friction units in aviation, ship, and mechanical engineering, etc. [1,2,3,4,5,6].

Considerable attention is paid to various methods of forming functional coatings on the surfaces of metal, ceramic, and polymer materials-based items [7,8,9,10]. An experimental assessment of modified polyethylene-based thin-film coatings was carried out in [11,12]. The biological, chemical, and tribological “usefulness” of surface modification was also demonstrated in [13,14,15,16].

There are several approaches to the formation of thin-film coatings (from gluing or applying a “ready-made” protective film to polymerization of twisted fluorinated monomers) [17,18,19]. Each approach is characterized by certain resource and energy intensities, environmental friendliness, ergonomics, and duration of the formation process, thus producing coatings with a range of physicochemical characteristics (wettability, permeability, strength, etc. [20,21,22,23]). And there are optimal conditions for the implementation of each modification technique to ensure that the best functional characteristics are achieved in the development of materials [24,25,26].

It is known that due to the peculiarities of chemical structure and morphology, the fluorinated polymers have low water and air permeability, electrical insulating properties, and high chemical and heat resistance [27,28,29,30,31]. The authors of [32,33,34] are devoted to the fluorination of polyethylene terephthalate, and the authors of [35,36] investigate polystyrene. The issues of fluorination of polyvinyltrimethylsilane and polyphenylene oxide were investigated in [37,38]. A method of selective modification of polymer films based on a combination of fluorination, sulfonation, and photolithography is presented in [12]. It is devoted to the creation of mosaic thin-film coatings for which the wettability depends on the topographic location of a liquid drop on the wetted surface. The original technologies of gas-phase and plasma-chemical modification are presented in [39,40,41]. They have been proven to be effective in providing chemical resistance and mechanical strength in a number of polymers with respect to shear tangential deformations [42,43].

A well-known alternative to additive thin-film coating formation is liquid-phase physical modification [44,45]. An example of its implementation is the deposition of nano- or submicron particles from colloidal solutions onto the surface of a substrate [46,47]. Carbon nanotubes (CNTs) are actively used as such particles [48,49]. The issues of CNT synthesis, property analysis, and practical applications are presented in [50,51,52]. But it seems that, for the field of application under consideration, particle deposition is unacceptable for the functional coating formation due to the low micromechanical strength of the structures being formed and the relatively high cost of such implementation. In addition, the adhesive contact of additive coatings with the substrate is almost always lower than in the case of coatings organically integrated with the volume of the material [53,54,55].

Thus, the methods for modifying the surface of polymer materials are highly effective and have been sufficiently studied. They allow us to create chemically resistant coatings that are integrated with polymer films and plates. This allows us to expect their successful application in the formation of integrated functional coatings on 3D-printed products.

Some approaches and methods for morphological kinetics modeling of surface modification processes of polymer products and corresponding integrated thin film coatings are presented in [56,57]. Particular fluorination kinetics models for polybuta-diene, polysulfone, and polycarbonate siloxane have been proposed and generalized in [58,59]. The influence of chemical structure and surface microrelief on adhesive and chemical properties of modified polymer layers was considered in [60,61,62].

The main techniques of the polymer materials chemical structure being studied are the IR-Fourier spectroscopy [63,64,65]) and scanning electron microscopy (in the mode of mapping the polymer matrix chemical elements’ distributions over the surface of the modified polymer layers) [66,67,68]. The nanorelief structure can be fixed by means of atomic force, scanning electron, and high-resolution optical microscopy [69,70,71]. An original technique for analyzing the corresponding images of the experimental samples’ surface in order to quantify the observed morphological heterogeneity is presented in [72,73,74].

The purposes of this work are the comprehensive study and the comparative analysis of the macroscopic texture design and the gas-phase fluorination technological parameter effect on the morphology and the energy state of the 3D-printed test products’ surface.

## 2. Materials and Methods

### 2.1. The Macroscopic Texture Design of 3D-Printed Products’ Surface

**Figure 1:**
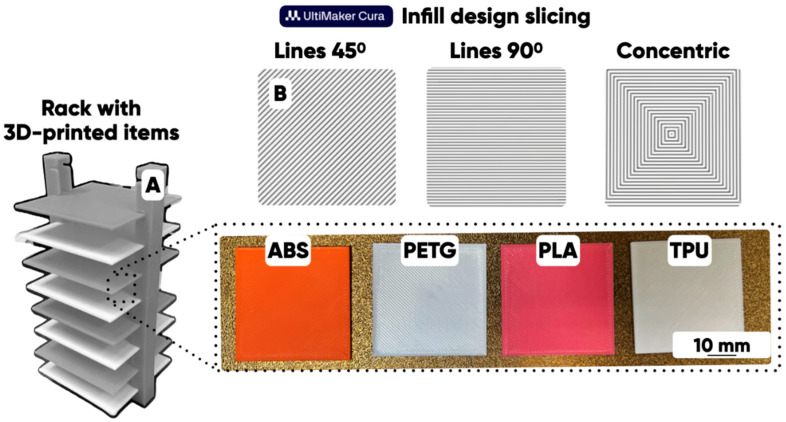
The ABS-, PETG-, PLA-, and TPU-filament-made test products (**A**) prototyped with the identical Anycubic Kobra Go FFF 3D printers and the texture design options, and (**B**) constructed using the UltiMaker Cura computer program (version 5.11-alpha) for 3D rasterization (slicing).

Twelve series of the experimental samples with 40 mm × 40 mm× 0.8 mm dimensions were produced to study the surface textural design (“linear”, “diagonal”, and “concentric”) effect on the properties of the 3D-printed products made of ABS, PLA, PETG, and TPU filaments using identical FFF 3D printers (Anycubic Kobra Go Shenzhen,, China) (Figure 1).

The values of the 3D printing technological parameters (the temperature of the dye, the temperature of the printing platform, and the 3D printing speed) used are shown in Table 1.

An increase in the 3D-printed products’ quality level (a decrease in the number of observed structural defects) can be achieved as a result of adjusting the Flow parameter at the prepress stage (during the 3D model-based STL file slicing) [43]. It was taken into account in this work during the 3D-printed products manufacturing (Figure 1A).

### 2.2. The Microscopic Texture Design of the 3D-Printed Products’ Surface

**Figure 2:**
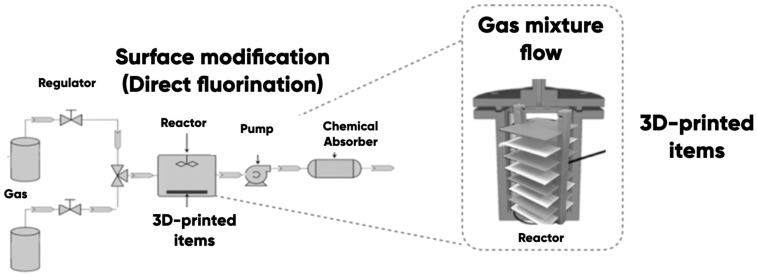
The scheme for 3D-printing product surfaces with the fluorine-containing gas mixture modification technique.

Surface properties of the polypropylene-based substrate and the 3D-printed test product’s direct control were carried out using the original gas-phase fluorination technique (Figure 2). A fluoro–helium mixture with a fluorine content of 13.5 vol.% (and 86.5 vol.% of helium) was used in accordance with the procedure [75] for the modification. The gas-phase fluorination duration was 0.25, 0.5, and 1.0 h.

During the preparation stage for modification, the surface of the samples was cleaned from contamination using a mechanical and chemical method (wiping with ethyl alcohol and drying under normal conditions for a day). The experimental samples were then placed into a reaction chamber and degassed by vacuum.

The fluorination of polymer products was carried out in a static mode. The samples were placed in a 5 L steel reactor. The fluoro-helium mixture $\left(He/F_{2}=86.5/13.5\;vol.\%\right)$ was introduced into the pre-vacuumed reactor chamber at the atmospheric pressure (10^5^ Pa) under the room temperature (20 ± 2 °C).

The gas-phase reaction products were removed by vacuuming and disposed of using standard chemical absorbers after the expiration of the time interval planned by the experimental program. Experimental samples were removed after the reactor chamber was de-vacuumed.

### 2.3. The 3D-Printed Products’ Surface Structure and Properties Studying

The experimental samples’ fluorination efficiency was characterized gravimetrically:

- The sample’s mass changing due to the gas-phase fluorination was determined using precision scales;
- The degree of fluorination $C_{A}^{F}\left(g/m^{2}\right)$ was calculated as a quotient of the induced mass increment by the sample’s surface area.

An empirical study of the experimental samples’ morphology was carried out using the scanning electron microscopy (SEM) (Jeol JSM 7500 (Tokyo, Japan)) and the infrared frequency range electromagnetic radiation absorption Fourier spectroscopy (IRFS) (Simex FT-801 IR spectrometer (Novosibirsk, Russia)) techniques.

**Figure 3:**
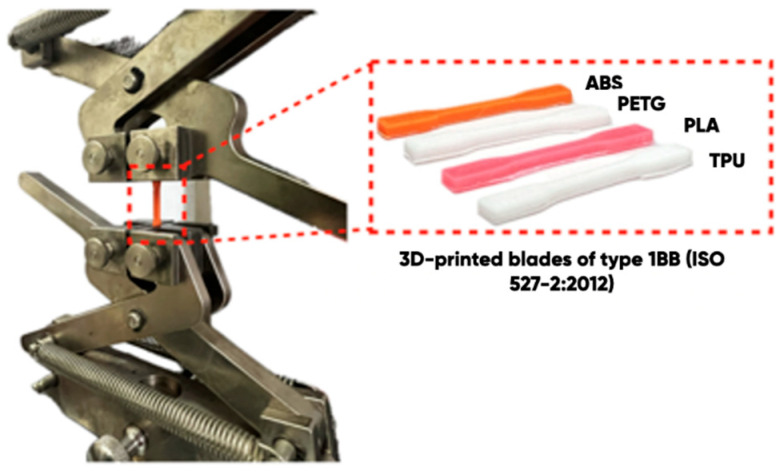
1BB-type 3D-printed blades.

Experimental samples’ surface wetting edge angles with the distilled water and the ethylene glycol were measured using the specialized KSVCAM 101 equipment (KSV Instruments, Helsinki, Finland) (Figure 3).

The specific surface free energy $\gamma_{S}\left(mJ/m^{2}\right)$ calculation (its polar $\gamma_{S}^{P}$ and disperse $\gamma_{S}^{D}$ components) was carried out using the Owens–Wendt–Rabel–Kaelble (OWRK) technique [76,77,78] taking into account the Girifalco–Good–Fowkes study [79,80,81,82].

The effect of gas-phase fluorination on the 1BB-type 3D-printed blades’ deformation strength was evaluated using a Zwick Roell BZ1.0 universal bursting testing machine. The straining friction coefficient was measured using a universal friction machine (MTU-01, Saint Petersburg, Russia).

### 2.4. The Variation–Rotational Maps Creation Technique

The digital SEM-image is formed as a tabular function $L\left(x,y\right)\;$ of pixels’ lightness L that depends on the corresponding coordinates $\left(x,y\right)$. This image can be cloned with the rotation to some angle φ. It is only necessary to calculate the coordinates as follows:

(1) $$\left(\begin{array}{c} {\tilde{x}}_{\varphi }\\ {\tilde{y}}_{\varphi } \end{array}\right)=\left(\begin{array}{cc} cos\varphi & sin\varphi \\ -sin\varphi & cos\varphi \end{array}\right)\cdot \left(\begin{array}{c} x\\ y \end{array}\right)$$

and to implement the assignment operation as follows: $\tilde{L}\left(x,y\right)=L\left({\tilde{x}}_{\varphi },{\tilde{y}}_{\varphi }\right)$.

One can obtain the distribution maps of the average values $\bar{L}\left(x,y\right)$, standard deviations $\sigma_{L}\left(x,y\right)$, and variation coefficients of $V_{L}\left(x,y\right)$, where

(2) $$\bar{L}\left(x,y\right)=\sum_{\varphi =\varphi_{1}}^{\varphi_{N}}L_{\varphi }\left(x,y\right)/N$$

(3) $$\sigma_{L}\left(x,y\right)=\sqrt{D_{L}\left(x,y\right)},\;D_{L}\left(x,y\right)=\sum_{\varphi =\varphi_{1}}^{\varphi_{N}}{\left(L_{\varphi }\left(x,y\right)-\bar{L}\left(x,y\right)\right)}^{2}/\left(N-1\right)$$

(4) $$V_{L}\left(x,y\right)=\sigma_{L}\left(x,y\right)/\bar{L}\left(x,y\right)$$

Averaged values of the variation coefficients should be used as the main quantitative character of the analyzed SEM-image rotational anisotropy.

## 3. Results and Discussion

### 3.1. The Surface Design Effect on the 3D-Printed Product Wettability

The 3D-printed products’ macroscopic surface design significantly affects their actual observed wettability (for PLA—31 $mJ/m^{2}$ [83], for TPU—34 $mJ/m^{2}$ [84], for ABS—33 $mJ/m^{2}$, and for PETG—38 $mJ/m^{2}$ [85]). Table 2 shows the results of measuring the surface wetting of the samples (with distilled water and ethylene glycol). Although the experimental samples were made from the same material, the values of specific valid free energy components differ significantly and depend on the direction in which the drop profile is projected (in a coordinate system determined by the sample surface texture) during measurement.

It should be noted that Table 2 shows the results of wetting angle measurements along the same direction in the laboratory coordinate system. Droplets on the surfaces under consideration are elongated along the 3D strokes and, as a result, the wetting angle depends on the direction of measurement. Macroscopic texturing provides actual wetting anisotropy due to differences between the surface of 3D-printed products and the plane. This anisotropy will not be visible to the naked eye in materials made of films.

But even the minimum observed wetting angle is large enough for the application of functional layers to the surface of test products, so an adhesive must be added to the ink composition.

The previously proven gas-phase fluorination technique [86] was applied to increase the wettability of the 3D-printed products surface. Corresponding results are presented in Table 3.

**Figure 4:**
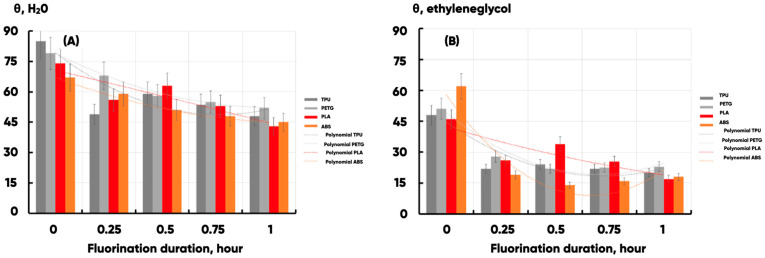
The water (**A**) and the ethylene glycol (**B**) wetting edge angle ($\theta_{H_{2}O}$ and $\theta_{C_{2}H_{6}O_{2}}$) dependencies on the fluorination procedure duration (T, hour) for the PETG-(light gray), TPU-(light dark gray), PLA-(red), and ABS-(orange) based samples. Second-order polynomials were used for the approximation in all cases.

It can be seen (Figure 4) that in all cases, the surface wettability of experimental samples increases significantly. However, this is accompanied by multidirectional changes in the components of the specific free surface energy and various types of induced transformations of the experimental sample surface energy state. The largest increase in the specific free surface energy was observed for PLA (from 35 to 55 $mJ/m^{2}$). It is explained by a more than fourfold increase in surface energy polar component ($\gamma_{S}^{P}$)—from 11 to 43 $mJ/m^{2}$. The change in the wettability of TPU is also mainly due to an increase in the polar component of the specific free surface energy, whereas for ABS and PETG, there is a significant increase in the one for dispersion (compared with the initial values).

It is known that the surface wettability is significantly affected by its chemical and physical structure (this follows from the possibility of appropriate Gibbs energy factorization). Changes in the chemical composition and surface structure of the filament during its curing are due to various factors, including the elemental composition, chemical structure, temperature and speed of extrusion, air (atmospheric) composition, and other conditions under which curing takes place. In particular, short-term exposure to elevated temperatures can lead to some oxidation of the surface, which contributes to hydrophilization.

### 3.2. The Effect of the Fluorination on 3D-Printed Product Surface Morphology

Obtained values of the modification degree $C_{A}^{F},g/m^{2}$ (calculated as the ratio of the sample mass change to its surface area) are significantly higher for the 3D-printed materials (Table 4) than for the large-tonnage polyolefins- (polyethylene, polypropylene, polyvinyl chloride, and polyethylene terephthalate) made films and for the elastomers- (butyl rubber, nitrile butadiene rubber, ethylene propylene triple rubber copolymer, etc.) based plates [61,87].

**Figure 5:**
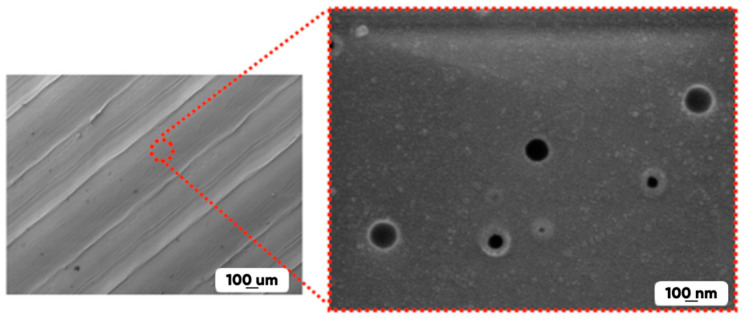
The multi-scale SEM-images of the PLA-made 3D-printed products’ surface.

The significant differences in the 3D-printed samples’ fluorination degree from ones for polymer material-made films and plates are due to the following:

(a) The presence of pores (with the diameters of 50 nm or more) in the initial and in the modified experimental samples structure contributing to the effective diffusion of the active reagent (fluorine) and significantly increasing the interaction with the modified gas mixture 3D-printed product surface area (Figure 5).
(b) The presence of unsaturated bonds in the 3D-printed polymers’ macromolecules (Table 5), which contribute to the intensive course of fluorine addition reactions.

**Figure 6:**
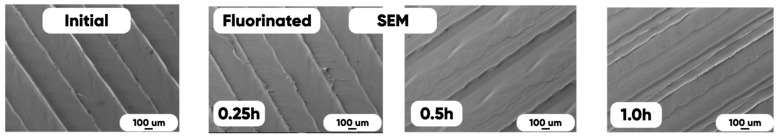
The SEM-images of the initial and the 0.25, 0.5, and 1.0 h fluorinated thermoplastic polyurethane samples.

The effect of the fluorination on the macroscopic structure of the 3D-printed products’ surface manifested itself in the form of distortion and/or disappearance of the boundaries between the adjacent 3D-strokes (Figure 6). It is probably caused by the thermally induced melting of the bulges with the largest specific area of active reagent contact (the fluorination and oxidation reactions are exothermic).

The parameters (for example, “period” and “borehole”) described the well-observed periodic elements of the 3D-prototypes’ macroscopic surface texture depending on the geometric design of the surface, the slicing settings, the diameter of the 3D-printer die, the extrusion speed of the filament, and the speed of 3D-printer’s printhead movement. The surface structure is formed as a result of the 3D-strokes coming into contact with each other.

The macroscopic texture of non-periodic elements, which are poorly observed with the naked eye at a macroscopic scale, depends more on the temperature of the filament, its melt fluidity index, and the curing rate, since they are the result of self-organization of the material structure after phase transition. They can also affect wettability due to changes in the true surface area. Thus, 3D-printed product macroscopic surface textures are mainly determined by relative position and 3D-stroke dimensions.

Thus, 3D-products’ actual surface areas are significantly larger than the areas of their projections onto the plane of the 3D printer’s object platform. And the degree of their difference from each other is determined by the sizes and the features of the relative arrangement of the fully macroscopic 3D-strokes that forming the 3D-prototypes following the “surface design”.

**Figure 7:**
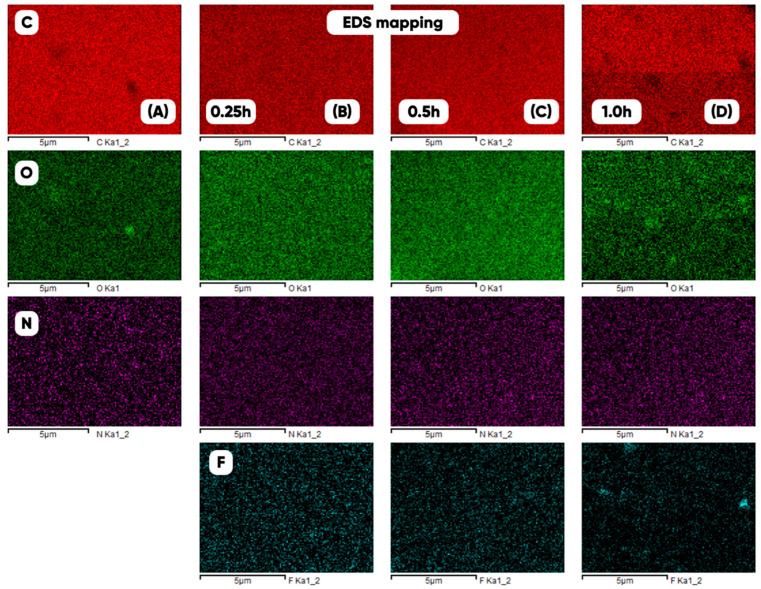
The planar distributions (EDS-mapping) of the carbon (C), oxygen (O), nitrogen (N), and fluorine (F) for the initial (**A**) and the fluorinated (0.25 (**B**), 0.5 (**C**), and 1.0 (**D**) h) 3D-printed products based on thermoplastic polyurethane.

At the microscopic level, the gas-phase modification by a fluorine-containing gas mixture had a significant effect on the nature of the planar distribution of the material-forming elements. Figure 7 shows the distribution maps of carbon, oxygen, nitrogen, and fluorine obtained by X-ray photoelectron spectrometry technique for the initial and the modified samples of thermoplastic polyurethane within 0.25, 0.5, and 1.0 h of gas-phase treatment. The carbon planar distribution actually repeats the surface microrelief.

A previously developed approach of variation–rotation patterns [39,42] was applied to quantify the modification influence on the chemo-morphological transformations of the experimental samples’ surface structure at the microscopic scale (Table 6). The essence of the method consists of cloning the source images with rotation by a certain angle (in our case by 15 degrees) and calculating main statistical characteristics (average value, standard deviation, and variation coefficient of lightness) for each pixel location in the resulting pattern.

The average value, standard deviation, asymmetry, and kurtosis of the pixel lightness variation coefficients, as well as the size of the chemo-morphological structure stability domain at levels 0.05 and 0.5, are presented in Table 7.

All obtained empirical statistical distributions are characterized by a sharper peak than normal ones and are described by an asymmetry in which the mode does not exceed the mathematical expectation. The planar carbon distribution (which largely determines the microtexture of the polymer matrix) is expected to have the highest structural stability (with the minimum average value and standard deviation of the pixel lightness coefficient of the variational–rotational pattern). The lowest structural stability (prior to the modification) is characteristic of the planar nitrogen distribution (the maximum mean and deviation of the coefficient of variation is $V_{N}≅0.9\pm 0.2$). The fluorine planar distribution becomes least uniform ($V_{F}≅1.2\pm 0.3$) and the variability of the nitrogen distribution decreases ($V_{N}≅0.7\pm 0.1$) after the long (1 h) tested sample modification. At the same time, the structural stability of the polymer matrix decreases ($V_{C}≅0.21\pm 0.05$) and the morphological heterogeneity of the oxygen distribution during the gas-phase treatment passes through a minimum value ($V_{O}≅0.23\pm 0.05$). Therefore, it is thus shown that gas-phase fluorination contributes to significant changes in the mode structure of roughness and material-forming elements on the surface of 3D-printed products at the microscopic level.

Results of the elemental analysis of changes in the atomic content of carbon, oxygen, nitrogen, and fluorine due to the gas-phase modification of TPU indicate the nonlinearity of the surface fluorination kinetics of the corresponding 3D-printed test products (Table 8).

The carbon content in the surface layer of the experimental samples (correlated with the macroscopic features of the microrelief) slightly decreases (at the level of statistical error) due to gas-phase processing. This corresponds to macroscopically observed “blur-ring” of the neighboring 3D-stroke boundaries.

**Figure 8:**
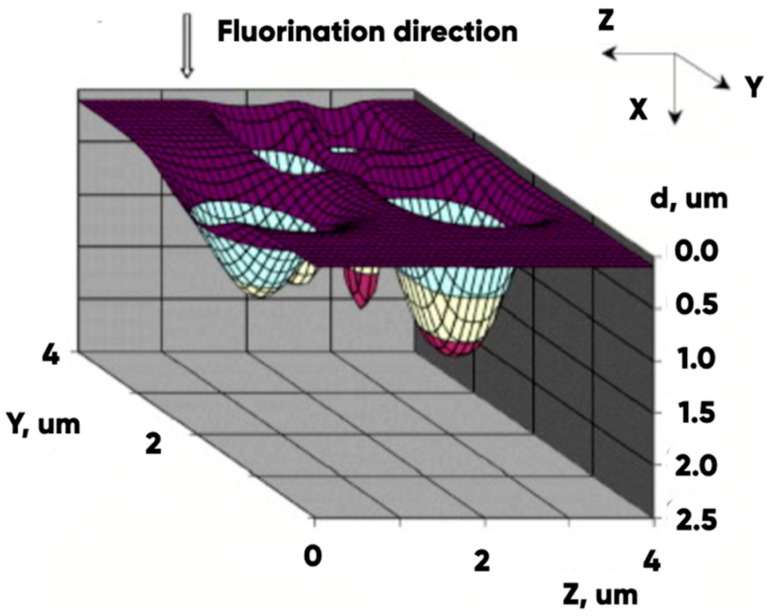
The typical result of polymer material fluorination dynamics simulation [56].

A detailed analysis of the dynamics of fluorination for a number of polymer materials using various approaches to simulation is presented in a large number of scientific papers, including some papers written by our research team previously. For example, ref. [56] showed that the distribution of fluorine in modified surface layers was significantly heterogeneous across the surface area, and nonlinear with respect to the depth of penetration of the modifier into the polymer matrix. (Figure 8).

Empirically obtained results of gravimetric measurements (Table 4) and EDS-analysis (Table 8) confirm the typical characteristic of the fluorination process, which is nonlinearity for the considered 3D-printed materials.

A decrease in the atomic oxygen content combined with an increase in the nitrogen content in the analyzed layer, most likely, indicates the destruction of NC bonds in TPU macromolecules accompanied by the addition of fluorine to nitrogen and carbon atoms. The nonlinearity of the fluorine content change is most likely due to the depletion of the number of CH- bonds in which the hydrogen atoms would not be replaced by the fluorine ones after a long (1 h) gas-phase modification.

**Figure 9:**
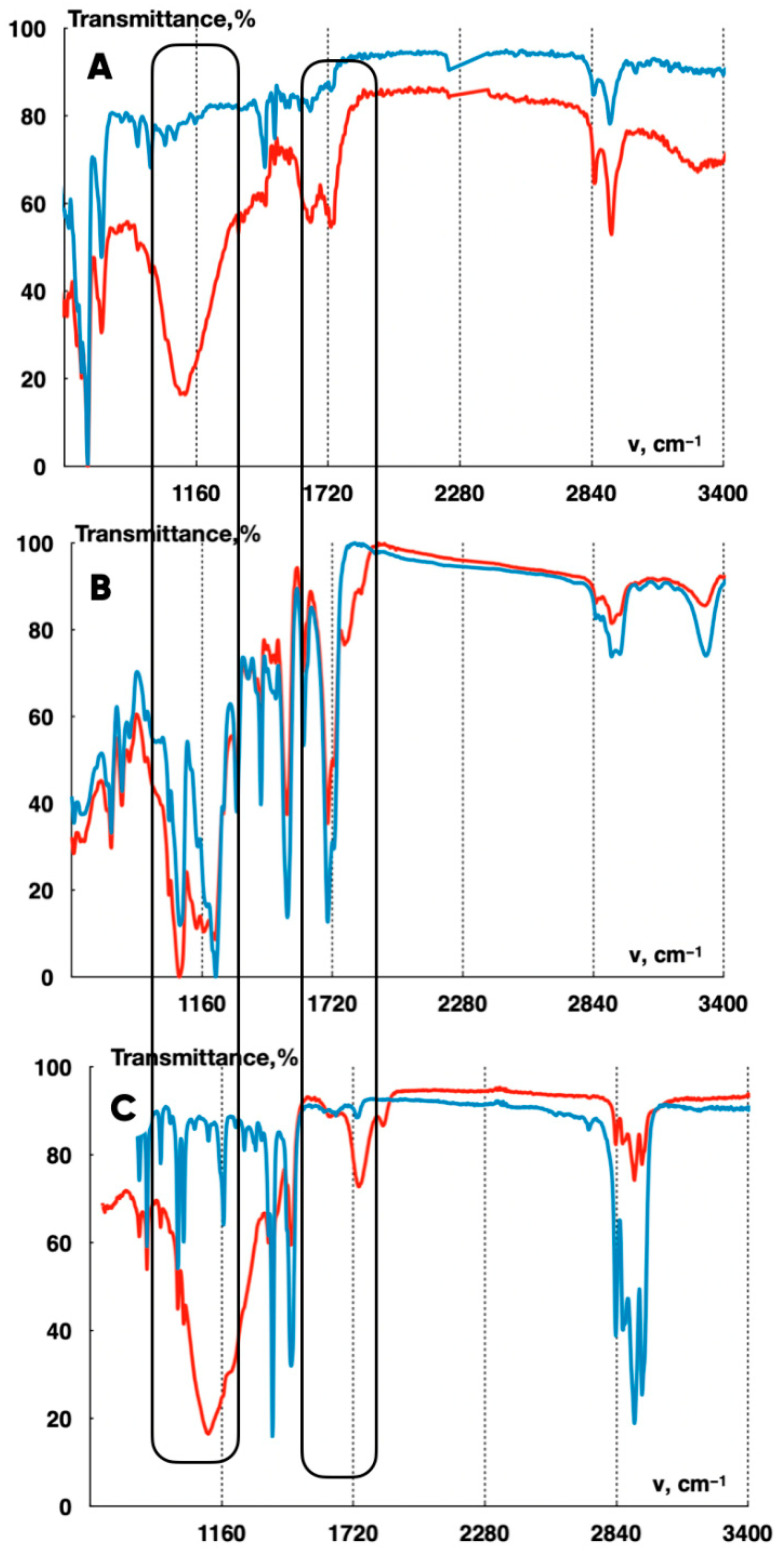
IR-Fourier spectra for the initial and the fluorinated 3D-printed products based on ABS (**A**), TPU (**B**), and polypropylene (**C**) (comparison sample). The blue color indicates the absorption spectra of the infrared electromagnetic radiation for the initial samples; the red indicates the fluorinated ones.

The significance of the fluorinated 3D-printed products’ surface chemical structure changes is also evidenced by the IR-Fourier spectroscopy results obtained for the ABS-, TPU-, and PP-made samples (Figure 9).

The IR spectra (Figure 9) show that all the considered types of polymers (ABS, TPU, and PP) are characterized by the appearance of a dip with the wave numbers 1100–1365 cm^−1^ corresponding to the valence vibrations of the CF-bonds. The difference in the intensity of the corresponding spectral lines is due to the peculiarities of the polymers’ chemical nature.

Chemical transformations of the polymer materials under consideration caused by their induced oxidation (Figure 9A,C) or by the changes in the character of CO-bonds (Figure 9B are accompanied with the increase (Figure 9A,C) or decrease (Figure 9B) in IR-radiation absorption in the 1600–1800 cm^−1^ wave numbers range.

The overall increase in the absorption (10–15%) of the infrared frequency range electromagnetic radiation in the ABS-based samples (Figure 9A) as a result of the fluorination indicates significant changes in the texture and the chemical structure of the corresponding products’ surface. The increase in the 2800–2900 cm^−1^ CH-bonds region (Figure 9A) is probably due to the chemo-induced destruction of double C=C bonds and the cycles opening. The decrease in the CH-bonds region (Figure 9B,C) is usually interpreted as a consequence of hydrogen substitution reactions with the fluorine.

The increase in the transmission (in 3000–3400 cm^−1^ range) (Figure 9B) indicates the destruction (as a result of fluorination) of OH-groups formed when the TPU filament comes into contact with the atmospheric moisture during the curing.

**Figure 10:**
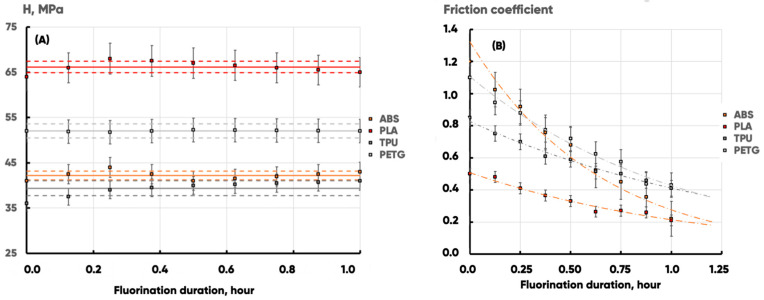
The experimental samples’ hardness (H, MPa) (**A**) and straining friction coefficient (μ, un.) (**B**) Dependences on the fluorination procedure duration (T, hour) for ABS (orange), PLA (red), PETG (dark gray), and TPU (light gray). The continuous and dashed lines indicate mean values and confidence intervals in (**A**). The dashed–dotted lines in B are the result of the experimental data exponential approximation.

It is well-known that polymer product fluorination under extreme conditions can lead to their destruction due to insufficient mechanical strength. It was found (Table 9) that the gas-phase fluorination under the experimental conditions does not affect the tensile strength of 3D-printed blades made of ABS, PLA, TPU, and PETG filaments (the values remain unchanged within the statistical error (Figure 10A).

At the same time, the strain-induced friction coefficient (maximum rest friction coefficient) significantly decreases, which correlates with an increase in abrasion resistance (Figure 10B). Microscopic transformations of the supramolecular structure of polymer materials caused by changes in the nature of material-forming element (carbon, oxygen, and to some extent nitrogen) planar distributions lead to improvements in the tribological properties of surfaces.

Thus, the demonstrated structural, morphological, and chemical transformations of the near-surface and surface layers of 3D-printed products as a result of macroscopic (3D printing) and microscopic (gas-phase modification) design provides the possibility of direct regulation of lyophilic, tribological, and other properties (depending on surface energy state).

## 4. Conclusions

The direct physico-chemical and textural design of the surface was carried out. Texture modeling was performed, and the properties of integrated submicroscopic coatings (obtained as a result of 3D printing of test products’ surfaces) were determined. The straining friction coefficient and wettability were experimentally determined.

The straining friction coefficient and wettability were experimentally determined. Interrelationships between microtexture, chemical composition, and structure and functional properties of additive-prototyping polymers were established. Absence of a statistically significant effect of fluorination on deformation strength of 3D-printed test products was demonstrated.

It is shown that the surface design of 3D-printed products promotes hydrophobization and anisotropy of wetting. The distilled water edge angle varies from 76 to 116 degrees for the same sample, simultaneously demonstrating hydrophilicity in one direction and hydrophobicity in the other.

It is established that the gas-phase surface modification technological regime (the duration of the material surface treatment with a fluorine-containing gas mixture) provides the possibility of FFF 3D prototyping products’ surface structure direct control at the micro-scale level.

Thus, the combination of the extrusion additive prototyping and the gas-phase surface modification techniques is the most promising way to form the functional (including bio- and chemo-resistant) surface coatings integrated with a polymer matrix volume.

The combination of developed analysis and modeling techniques will allow us to further formulate theoretical descriptions of high-innovation potential technologies for directly controlling the functional properties of bulk modified polymers used in 3D printing.

Such materials can be used in construction, when creating water-resistant materials and sealing products; in medicine, when creating biocompatible products, including biodegradable and bio-resistant ones; and in the automobile industry, such as in panel, chassis elements, suspension, intake manifold, valve cover, and turbocharger housing manufacturing.

Typical limitations of polymer composite use in industry include an insufficiently high level of mechanical strength, low temperature resistance, flammability and toxicity of combustion products, and the complexity of product molding. The advantage of gas-phase fluorination exploitation is the possibility of carrying out the functionalizing finishing surface treatment of 3D-printed items manufactured using already developed and well-proven additive prototyping techniques.

## Figures and Tables

**Table 1:** The 3D printing technological parameters and filament polymer types used when the experimental samples additive prototyping.

Type of Polymer (Filament)	Manufacturer	3D Printing Options		
		Nozzle Temperature, °C	Printing Platform Temperature, °C	Printing Speed, mm/min
Acrylonitrile, butadiene and styrene copolymer (ABS)	Shenzhen Esun Industrial Co., Shenzhen, China	250	80	40
Polylactide (PLA)		210	50	40
Polyethylene Terephthalate Glycol (PETG)		235	60	40
Thermopolastic polyurethane (TPU)	U3print, Moscow, Russia	210	60	30

**Table 2:** Distilled water and ethylene glycol wetting edge angles and the corresponding values of the specific free surface energy (total, dispersion, and polar components) for the PLA-based 3D-printed samples at different macroscopic surface design (filament strokes orientation).

3D-Printed Product Surface Macroscopic Textural Design	Wetting Edge Angle, °		$\gamma ,mJ/m^{2}$	$\gamma_{S}^{D},mJ/m^{2}$	$\gamma_{S}^{P},mJ/m^{2}$
	$\theta_{H_{2}O}$	$\theta_{C_{2}H_{6}O_{2}}$			
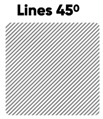	74 ± 7	46 ± 6	35 ± 4	24 ± 2	11 ± 2
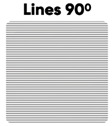	76 ± 8	59 ± 6	31 ± 3	12 ± 1	16 ± 2
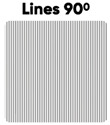	116 ± 8	91 ± 8	19 ± 3	18 ± 3	1 ± 1
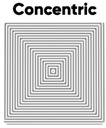	106 ± 8	65 ± 7	52 ± 5	51 ± 1	1 ± 1

**Table 3:** The distilled water and ethylene glycol wetting edge angles and the corresponding values of the specific free surface energy (total, dispersion, and polar components) for the TPU-, ABS-, PLA-, and PETG-made 3D-printed samples after the fluorination at different durations.

3D-Printed Product Surface Macroscopic Textural Design	Filament Type	Wetting Edge Angle, °		$\gamma ,mJ/m^{2}$	$\gamma_{S}^{D},mJ/m^{2}$	$\gamma_{S}^{P},mJ/m^{2}$
		$\theta_{H_{2}O}$	$\theta_{C_{2}H_{6}O_{2}}$			
		**Fluorination Duration (0 h)**				
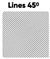	TPU	85 ± 9	48 ± 5	42 ± 4	40 ± 4	2.0 ± 0.5
	ABS	67 ± 7	62 ± 6	37 ± 4	3.2 ± 0.5	35 ± 4
	PETG	79 ± 8	51 ± 5	41 ± 4	8.0 ± 0.8	33 ± 4
	PLA	74 ± 7	46 ± 5	35 ± 4	24 ± 2	11 ± 2
	Fluorination duration (0.25 h)					
	TPU	49 ± 5	22 ± 2	50 ± 5	15 ± 2	36 ± 4
	ABS	59 ± 6	19 ± 2	46 ± 5	27 ± 3	19 ± 2
	PETG	68 ± 7	28 ± 2	44 ± 4	34 ± 3	10 ± 2
	PLA	56 ± 6	26 ± 2	45 ± 4	19 ± 2	26 ± 3
	Fluorination duration (0.5 h)					
	TPU	59 ± 6	24 ± 2	44 ± 5	24 ± 3	20 ± 2
	ABS	51 ± 5	14 ± 2	49 ± 5	20 ± 2	29 ± 3
	PETG	58 ± 6	22 ± 2	45 ± 5	24 ± 3	21 ± 2
	PLA	63 ± 6	34 ± 3	41 ± 4	21 ± 2	19 ± 2
	Fluorination duration (1 h)					
	TPU	48 ± 5	20 ± 2	51 ± 5	15 ± 2	36 ± 4
	ABS	45 ± 5	18 ± 2	53 ± 5	13 ± 2	40 ± 4
	PETG	52 ± 5	23 ± 2	33 ± 4	25 ± 3	7.7 ± 0.8
	PLA	43 ± 4	17 ± 2	55 ± 6	12 ± 1	43 ± 4

**Table 4:** The ABS-, PLA-, TPU- and PETG-made 3D-printed test products’ fluorination degree for different durations of gas-phase processing.

Modification Duration, h	$3\mathrm{D-Printed}\;\mathrm{Products}’\;\mathrm{Fluorination}\;\mathrm{Degree}\;C_{A}^{F},g/m^{2}$			
	ABS	PLA	TPU	PETG
0.25	4.1 ± 0.4	2.8 ± 0.2	2.3 ± 0.2	5.3 ± 0.4
0.5	17.5 ± 0.8	7.0 ± 0.5	8.0 ± 0.5	7.3 ± 0.5
1.0	21.9 ± 0.8	13.3 ± 0.5	22.2 ± 0.8	13.0 ± 0.5

**Table 5:** The chemical structure of 3D-printed materials under consideration.

Abbreviation	Chemical Formula
ABS [88]	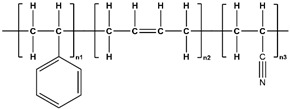
PLA [89]	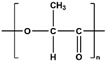
PETG [90]	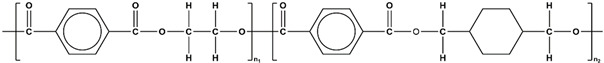
TPU [91]	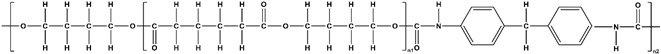

**Table 6:** Frontal and profile projections of the variation–rotational maps constructed for the planar distributions (Figure 7) of carbon, oxygen, nitrogen, and fluorine. The square side length is ~5 microns.

	Fluorination Duration, Hour			
	Initial	0.25	0.5	1.0
C	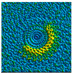	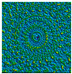	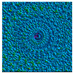	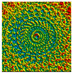
	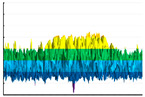	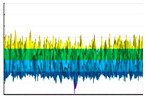	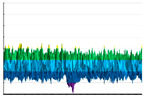	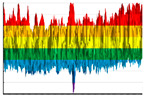
O	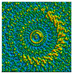	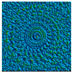	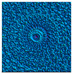	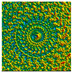
	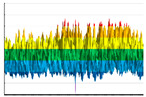	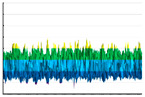	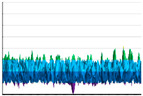	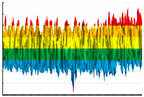
N	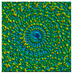	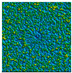	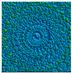	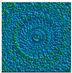
	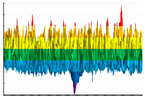	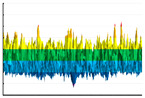	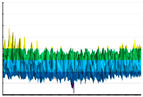	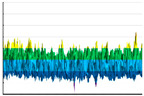
F	-	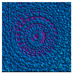	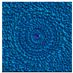	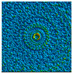
		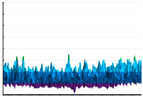	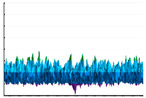	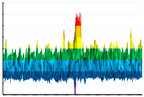

**Table 7:** Quantitative characteristics of the variation–rotation patterns formed as a result of the material-forming element (carbon, oxygen, nitrogen, and fluorine) planar distribution (Figure 6) computer analysis.

Gas-Phase Fluorination Duration, Hour	0.00	0.25	0.5	1.0
**Carbon Planar Distribution Quantitative Characteristics**				
Average value of the variation coefficient	0.13	0.14	0.12	0.21
Standard deviation of the variation coefficient	0.04	0.03	0.03	0.05
Asymmetry of the variation coefficient distribution	0.77	0.37	0.28	0.36
Kurtosis of the variation coefficient distribution	0.87	0.34	0.25	0.07
Structural domain characteristic size (at 0.05 level), nm	120	130	290	60
Structural domain characteristic size (at 0.5 level), nm	5000	5000	5000	5000
**Oxygen planar distribution quantitative characteristics**				
Average value of the variation coefficient	0.40	0.29	0.23	0.47
Standard deviation of the variation coefficient	0.09	0.07	0.05	0.11
Asymmetry of the variation coefficient distribution	0.53	0.34	0.32	0.29
Kurtosis of the variation coefficient distribution	0.46	0.14	0.29	0.05
Structural domain characteristic size (at 0.05 level), nm	40	40	100	40
Structural domain characteristic size (at 0.5 level), nm	4640	4980	4990	3930
**Nitrogen planar distribution quantitative characteristics**				
Average value of the variation coefficient	0.89	0.78	0.66	0.66
Standard deviation of the variation coefficient	0.19	0.16	0.14	0.14
Asymmetry of the variation coefficient distribution	0.49	0.50	0.38	0.39
Kurtosis of the variation coefficient distribution	0.78	0.67	0.64	0.34
Structural domain characteristic size (at 0.05 level), nm	50	40	40	40
Structural domain characteristic size (at 0.5 level), nm	510	830	1710	1760
**Fluorine planar distribution quantitative characteristics**				
Average value of the variation coefficient	-	0.70	0.87	1.16
Standard deviation of the variation coefficient	-	0.20	0.19	0.29
Asymmetry of the variation coefficient distribution	-	0.78	0.57	1.13
Kurtosis of the variation coefficient distribution	-	1.06	0.90	3.43
Structural domain characteristic size (at 0.05 level), nm	-	40	40	60
Structural domain characteristic size (at 0.5 level), nm	-	1940	550	120

**Table 8:** Results of the elemental analysis of the carbon, oxygen, nitrogen, and fluorine content (excluding hydrogen) in the surface layer of a cured gas-phase-modified thermoplastic polyurethane filament.

Fluorination Duration, Hour	C (at. %)	O (at. %)	N (at. %)	F (at. %)
0	67.0 ± 0.8	25.0 ± 0.8	8.0 ± 0.1	-
0.25	66.0 ± 0.7	24.0 ± 0.7	8.0 ± 0.1	2 ± 0.1
0.5	66.0 ± 0.7	23.0 ± 0.6	7.0 ± 0.1	4 ± 0.2
1	66.0 ± 0.5	18.0 ± 0.4	10.0 ± 0.2	6 ± 0.4

**Table 9:** The hardness H (MPa) and the straining friction coefficient μ for the 3D-printed type 1BB tubes before and after the gas-phase fluorination.

Fluorination Duration, Hour	3D-Printing Material							
	ABS		PLA		TPU		PETG	
	H, MPa	μ	H, MPa	μ	H, MPa	μ	H, MPa	μ
0	41 ± 3	1.20 ± 0.10	64 ± 6	0.50 ± 0.05	36 ± 3	0.85 ± 0.08	52 ± 5	1.10 ± 0.10
0.25	44 ± 4	0.92 ± 0.09	68 ± 7	0.41 ± 0.05	39 ± 4	0.70 ± 0.07	52 ± 4	0.88 ± 0.09
0.5	41 ± 3	0.68 ± 0.07	67 ± 7	0.33 ± 0.04	40 ± 4	0.59 ± 0.06	52 ± 3	0.72 ± 0.08
1	43 ± 3	0.22 ± 0.02	65 ± 6	0.21 ± 0.02	41 ± 4	0.41 ± 0.04	52 ± 4	0.43 ± 0.04

## Data Availability

The original contributions presented in this study are included in the article. Further inquiries can be directed to the corresponding authors.
